# Supplementary material for: The Proteasome Inhibitor Bortezomib Induces Apoptosis and Activation in Gel-Filtered Human Platelets
Source: Int J Mol Sci. 2021 Aug 19;22(16):8955. doi: 10.3390/ijms22168955 (PMC8396574; doi:10.3390/ijms22168955)
Supplement: Supplementary file 1 [file ijms-22-08955-s001.zip › ijms-1319539-supplementary.pdf]

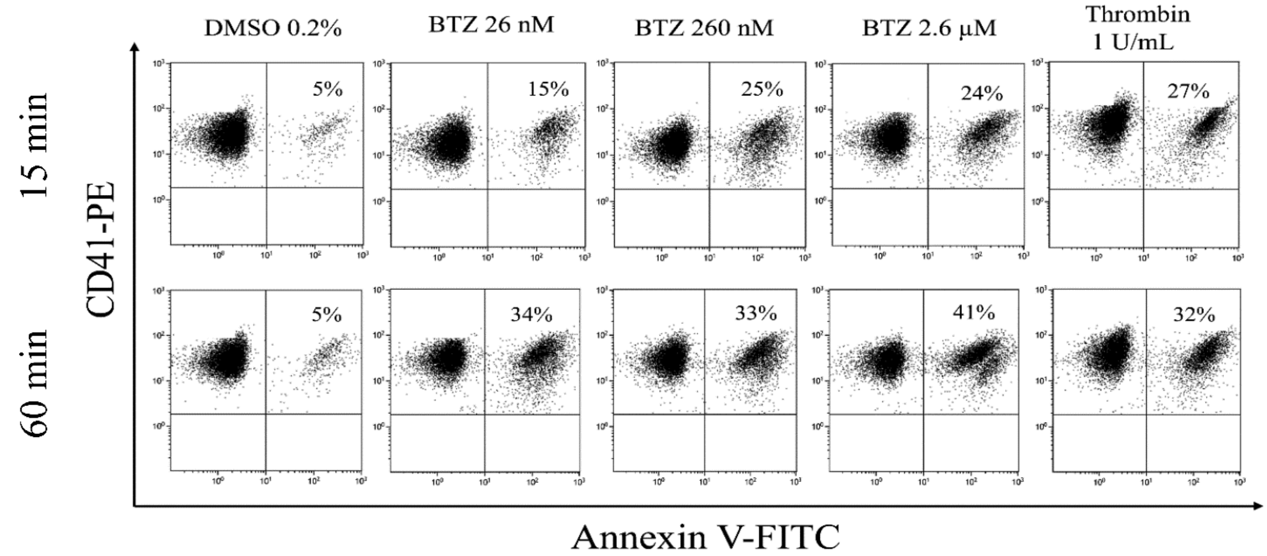

Figure S1. PS expression in BTZ-preincubated human GFP (Related to Figure 1B). GFP were treated with BTZ, thrombin (positive control) and DMSO (negative control) for 15 and 60 minutes. PS expression was measured by flow cytometry based on CD41 and Annexin V double positivity (upper right quadrant). Representative dot plots of the percentage PS expression of 1 of 6 experiments are shown. BTZ: bortezomib; DMSO: dimethyl sulfoxide.

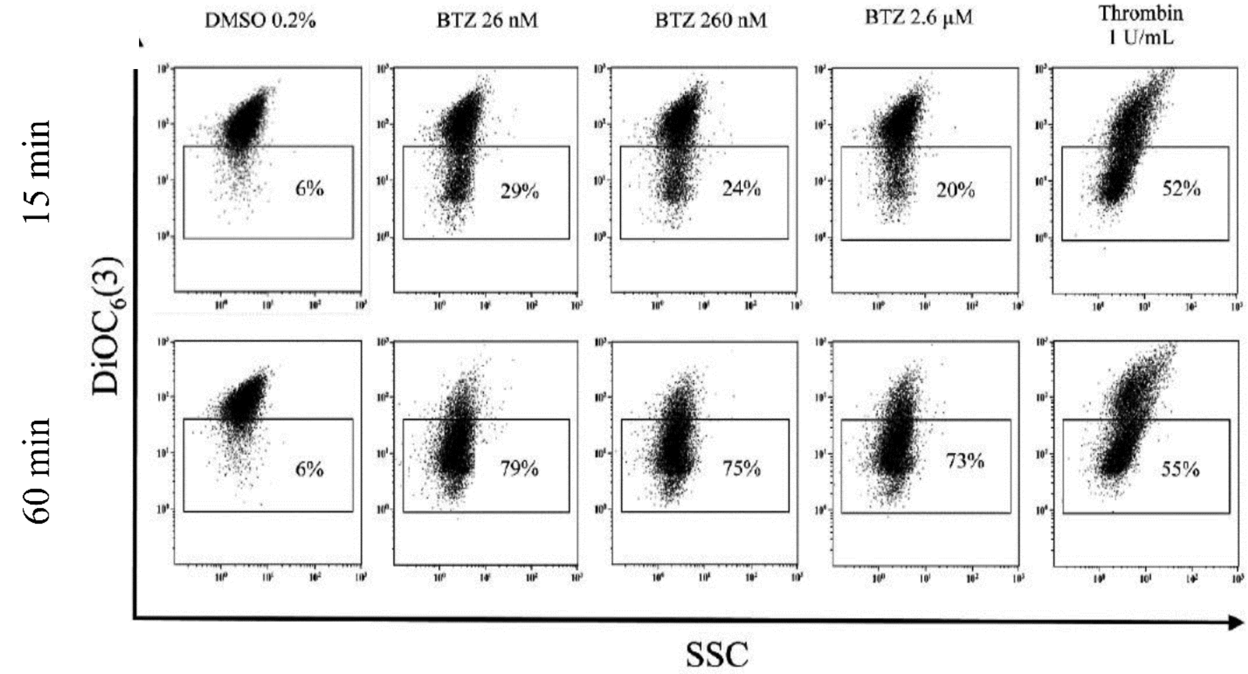

Figure S2. BTZ induces ΔΨm depolarization of human GFP (Related to Figure 3A). GFP were incubated with BTZ, thrombin (positive control) and DMSO (negative control) for 15 and 60 minutes. Depolarization of ΔΨm was analyzed by flow cytometry using $DiOC_6(3)$ dye. Representative dot plots of the percentage depolarized cells of one of twelve experiments are shown. BTZ: bortezomib; DMSO: dimethyl sulfoxide.

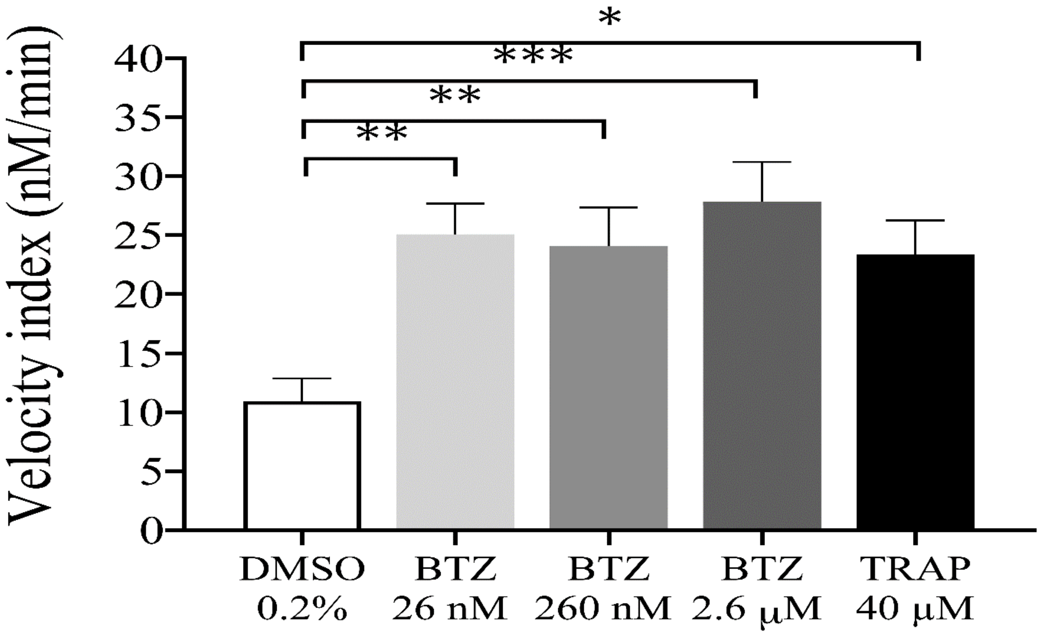

Figure S3. BTZ enhances *in vitro* thrombin formation in human GFP (Related to Figure 4). GFP were treated with BTZ, TRAP (positive control) and DMSO (negative control) for 60 minutes, and resuspended in autologous PPP to a final concentration of $20x10^9$/L. Thrombin generation was initiated with 1 pM rTF (PRP reagent). Mean ± SEM of 10 experiments for DMSO and BTZ or 5 experiments for TRAP is presented. The level of significance was determined using ANOVA, Dunnett's multiple comparisons test, *p<0.05, **p<0.01, and ***p<0.001 compared to the DMSO group. BTZ: bortezomib; DMSO: dimethyl sulfoxide; TRAP: thrombin receptor-activating peptide.

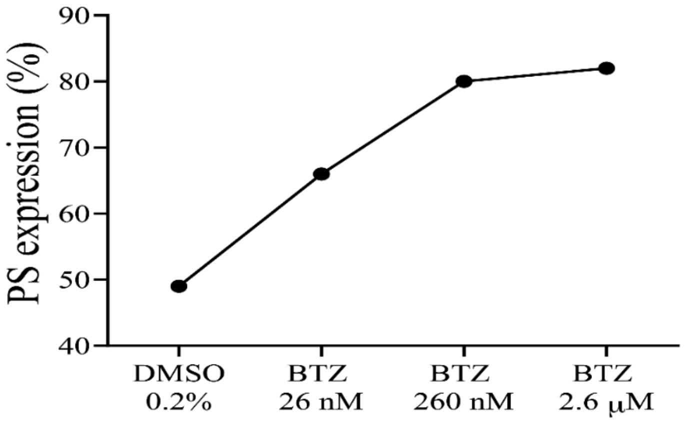

Figure S4. BTZ induces PS expression in peripheral blood B-cells. Magnetically isolated B-cells of a healthy donor was cultured with BTZ for 24 hours at 37 °C and 5% CO_2. The cells were washed by centrifugation at 1200 rpm for 5 min at RT and resuspended in 50 μl of Annexin V-binding buffer. The samples were stained with 5 μl of Annexin V-FITC, incubated for 15 min at RT in the dark, and diluted to 550 μl with Annexin V-binding buffer. PS expression was measured using an FC 500 flow cytometer (Beckman Coulter, Brea, CA, USA). BTZ: bortezomib; DMSO: dimethyl sulfoxide; PS: phosphatidylserine.

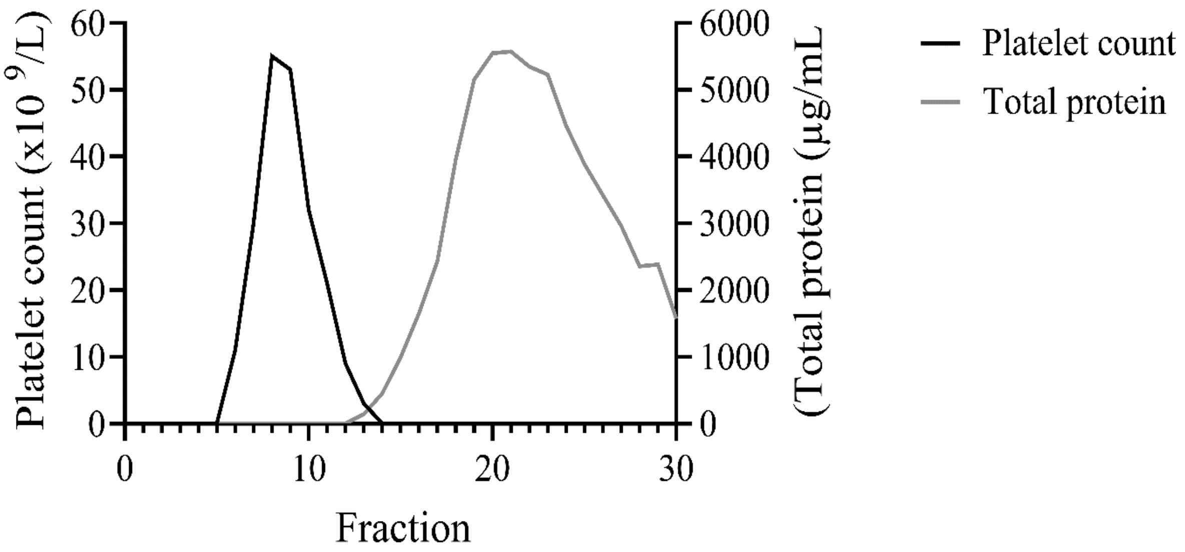

Figure S5. Determination of protein levels using the Pierce BCA protein-assay. In a preliminary experiment, platelets were isolated via gel-filtration chromatography and thirty fractions were collected into Eppendorf tubes. The platelet count of each fraction was determined by the Sysmex XP-300 hematology analyzer (Sysmex, Kobe, Japan) prior to centrifugation at 1500 g for 15 min at RT to sediment the cells. The presence of proteins in the supernatants were determined by the Pierce BCA protein-assay, following the manufacturer's instructions and the total protein concentrations were measured using a microplate reader (LabSystems MULTISKAN MS microplate reader). Platelet fractions totally devoid of proteins (as shown above) were used in subsequent experiments.
